# Development and validity of an educational booklet on LGBT+ health in Primary Health Care

**DOI:** 10.1590/0034-7167-2024-0433

**Published:** 2025-11-21

**Authors:** Milka Gabrielle de Lira Nóbrega West, Ednaldo Cavalcante de Araújo, Cláudia Margareth de Lira Nóbrega Vilar, Maria Amanda Lima Batista, Danilo Martins Roque Pereira

**Affiliations:** I Universidade Federal de Pernambuco. Recife, Pernambuco, Brazil; II Governo do Estado de Pernambuco, Secretaria de Saúde. Recife, Pernambuco, Brazil

**Keywords:** Sexual and Gender Minorities, Educational Technology, Continuing Education, Validation Study, Primary Health Care., Minorías Sexuales y de Género, Tecnología Educacional, Educación Continua, Estudio de Validación, Atención Primaria de Salud.

## Abstract

**Objectives:**

to develop and validate a digital educational booklet on LGBT+ health for professionals in Primary Health Care.

**Methods:**

a methodological study, prepared through bibliographic survey and analysis of the sociodemographic, educational and cultural profile of Primary Health Care professionals, content, appearance and semantics validity. For validity, the Content Validity Index equal to or greater than 0.80, binomial test and Suitability Assessment of Materials were adopted.

**Results:**

the booklet is composed of four axes: concepts on sexual and gender diversity; LGBTphobia; barriers to LGBT+ people’s health; and policies and legislation on LGBT+ people. Regarding content and appearance validity and semantic assessment, the booklet achieved a minimum Content Validity Index of 0.97.

**Conclusions:**

the booklet was considered valid and can be used as a tool for continuing education of healthcare professionals. Studies should be carried out to verify its effectiveness.

## RESUMEN

**Objetivos:**

desarrollar y validar una cartilla educativa digital sobre salud LGBT+ para profesionales de Atención Primaria de Salud.

**Métodos:**

estudio metodológico, elaborado mediante levantamiento bibliográfico y análisis del perfil sociodemográfico, educativo y cultural de los profesionales de Atención Primaria de Salud, validación de contenido, apariencia y semántica. Para la validación se adoptó el Índice de Validez de Contenido igual o mayor a 0,80, prueba binomial y la *Suitability Assessment of Materials*.

**Resultados:**

el folleto está compuesto por cuatro ejes: conceptos sobre diversidad sexual y de género; LGBTfobia; barreras a la salud para las personas LGBT+; y políticas y legislación sobre las personas LGBT+. En cuanto a la validación de contenido y apariencia y la evaluación semántica, el folleto alcanzó un índice de validación de contenido mínimo de 0,97.

**Conclusiones:**

el folleto fue considerado válido y puede ser utilizado como herramienta para la educación continua de los profesionales de la salud. Se deben realizar estudios para verificar su eficacia.

## INTRODUCTION

Lesbian, gay, bisexual, transvestite, transsexual and other forms of gender identity and/or sexual orientation (LGBT+) people, as they make up a group that diverges from heteronormative rules, have been scientifically called sexual and gender minorities, and are wrongly judged as uniform, when the multiple and complex experiences lived and associated with the intersectionality factors related to them are disregarded, such as employment, income, education and race/color^(1,2)^. Although they have representatives in various social spheres, such groups face a collective dynamic of insults, stigmas and violence related to gender identities that do not correspond to previously designated expressions, behaviors and social roles, or to a sexual orientation other than heterosexuality^(3)^.

LGBT+ people face several barriers to accessing healthcare services, including healthcare professionals’ attitudes and behaviors. Healthcare professionals often express negative or LGBTphobic attitudes. Therefore, there are many obstacles to communication, since it is not uncommon for this population group to be marginalized in healthcare services due to the use of offensive, violent language through derogatory words, jokes, verbal abuse, LGBTphobic gestures, use of pronouns and asking questions that invalidate gender identity and sexual orientation, especially for transvestite and transgender people who often do not even have the right to have their social name respected^(4,5)^.

Another barrier to access to healthcare is the lack of knowledge and awareness among healthcare professionals about the LGBT+ population’s specific needs. This gap is present in both training curricula and continuing health education programs, which often contain little or no information about sexual orientation and gender identity^(6)^. Furthermore, the visibility of the topic “LGBT health” in the scientific literature is limited^(7)^. Consequently, professionals commonly assume - wrongly - that LGBT+ people’s health needs are homogeneous, and therefore disregard singularities and do not always know how to adequately track which vulnerabilities are most frequently imposed for illness and provide adequate guidance on their healthcare^(2,4,5)^.

Primary Health Care is the gateway to care, and one of its goals is to improve access, which must be universal, in addition to providing comprehensive care to users and communities, based on identifying the social and health demands and needs of people and groups within the scope of action of Family Health teams (FHts). However, most of the time, such actions and plans do not consider the LGBT+ population as users of services and people with specific needs that determine and condition their health situation^(8-10)^.

Healthcare professionals must welcome, understand and assist in a respectful and decisive manner LGBT+ people’s specific health demands. However, there is a weakness in the care provided to them, caused by historical conceptions and definitions, and cultural aspects, still imbued with taboos and prejudices, influenced by the lack of information about such populations’ health demands, which permeates academic training and professional practices. Due to the continuous and disproportionate exposure to stigma-related stress, LGBT+ people avoid seeking healthcare services, or when they do seek them, they do not reveal their sexual orientation and/or gender identity as a protective measure to avoid suffering prejudice, which makes it impossible to identify specific needs, provide decisive assistance and establish a line of care^(1,5,10,11)^.

In order to transform work processes in healthcare services, where healthcare must be centered on people’s needs, healthcare professionals must be trained to care for LGBT+ people’s health, aiming at quality of care and their lives. Thus, it is necessary to expand the use of educational technologies that help professionals become prepared to welcome, establish bonds and provide comprehensive care to this population^(10,12)^.

An educational booklet focused on healthcare of LGBT+ people can be used as an important strategy to promote the process of acquiring, using and deepening knowledge, mastering skills and developing attitudes and autonomy that will influence decision-making and the implementation of health promotion actions by primary care professionals, addressing, in a succinct, clear and simple manner, topics related to the basic concepts of health issues of LGBT+ people and how to best serve them in Primary Health Care. Thus, the booklet can support healthcare practices in primary care, from the perspective of sexual and gender diversity, by seeking to promote possibilities for building equity and comprehensiveness in health, a principle of the Brazilian Health System (In Portuguese, *Sistema Único de Saúde* - SUS), and making health practices based on humanization.

## OBJECTIVES

To develop and validate an educational booklet on LGBT+ health for Primary Health Care professionals.

## METHODS

### Ethical aspects

The study was conducted in accordance with national and international ethics guidelines, approved by the *Universidade Federal de Pernambuco* Research Ethics Committee (REC) attached to this submission. The Informed Consent Form (ICF) was obtained from all those involved in the study online, using the electronic form tool Google Forms^®^.

### Study design, period and stages

This is a methodological study, developed from September 2023 to May 2024, consisting of the following stages: 1) project preparation and submission to the REC; 2) search in specialized literature; 3) development of educational material; 4) material validity by a committee of experts and assessment by the target audience. This study was structured in accordance with the Standards for QUality Improvement Reporting Excellence 2.0^(13)^.

In the first stage, the study project was prepared and submitted to the REC. The second stage involved a literature review based on scientific articles and information from the Brazilian National Policy for Comprehensive Health for Lesbian, Gay, Bisexual, Transvestite and Transgender People, in addition to an integrative review study^(6)^. The third stage consisted of developing the educational booklet content and layout, based on content extracted from literature and data on content and practice in LGBT+ health during the training and professional activity of Primary Health Care professionals, obtained through a semi-structured sociodemographic questionnaire, which included the question “What do you want to learn about LGBT+ health?”. The material produced was sent for formatting and configuration by a design professional for layout. Figma^®^ software was used for graphic creation. The fourth stage involved booklet validity by specialists in LGBT+ health.

### Sample; inclusion and exclusion criteria

Healthcare professionals were sought through the Brazilian National Council for Scientific and Technological Development *Lattes* Platform using the keywords “LGBT+ people health” and also through non-probabilistic snowball sampling, from which the *Lattes* CVs were assessed to verify whether professionals met the following inclusion criteria, considering Jasper’s precepts^(14)^: having knowledge/skills in LGBT+ health; professional experience in LGBT+ health; and expertise in studies on the development of educational technologies in health.

Eighty-one potential judges with doctoral, master’s and/or specialist degrees and professional experience in LGBT+ health from different states and all regions of Brazil were invited by email. Of these, 58 did not return the contact, and one judge did not completely complete the assessment instruments, resulting in a sample of 22 judges^(15)^.

The judges who agreed to participate in the study were sent the ICF, the booklet and the instruments for assessing content (Instrument for Validity of Educational Content in Health (In Portuguese, *Instrumento de Validação de Conteúdo Educativo em Saúde* - IVCES))^(16)^ and appearance (Instrument for Validity of Appearance of Educational Technologies in Health (In Portuguese, *Instrumento de Validação de Aparência de Tecnologias Educacionais em Saúde* - IVATES))^(17)^. A ten-day deadline was set for assessing the booklet and completing the instruments. Each topic in the instruments contained affirmative sentences about the items, and, after analyzing the booklet, judges could assess the item as follows: very adequate; adequate; partially adequate; and inadequate.

Space was also included for suggestions to be made by judges. The suggestions were organized and analyzed according to the variables of instruments (booklet objective, structure/presentation and relevance) and duly accepted. After suggestions and analysis by experts, changes were made to the educational booklet material with the help of a professional designer, which resulted in the booklet entitled “Knowing to care. Educational booklet on LGBT+ health for Primary Health Care professionals”, available through the link https://www.ufpe.br/documents/39790/2852686/CARTILHA+EDUCACIONAL+CONHECER+PARA+CUIDAR.pdf/16b26753-5fe8-44e2-8911-1e141749d387

The target audience was selected by non-probabilistic snowball sampling, consisting^(18)^ of 12 nurses who work in the FHt. Nurses were chosen as the target audience because nurses are, for the most part, the first professional with a university degree that users have contact with in primary care services. In the various fields of activity, nursing consultation stands out, whose implementation of the nursing process is related to collecting the history, making nursing diagnosis and the appropriate care plan, based on the understanding of the social and health context of LGBT+ people. Another area that requires emphasis is the performance by nurses of educational practices, with qualified information dissemination, with people and communities in Basic Health Units, schools and in other spaces in the territory assigned to Family Health Units, a contributing factor to raising awareness and raising the population’s awareness^(19)^.

For semantic validity, nurses individually assessed the digital booklet. A kit containing the booklet in PDF format and the Suitability Assessment of Materials (SAM) instrument^(20)^ was sent by email, to be returned within five days.

### Analysis of results, and statistics

For data analysis, a database was created in the EPI INFO version 3.5.4, in which validity was performed by double entry to compare discrepancies and correct errors in the data obtained by the assessment of expert judges and the target audience. After validity, the database was exported to the Statistical Package for the Social Sciences version 18.0, where analysis was performed. To characterize the personal profile, professional profile, experience and publication of judges participating in the research, and to characterize the personal profile, professional profile and nurses’ knowledge about LGBT+ people’s health and about national policies and qualifications for care of LGBT+ people, percentage frequencies were calculated and frequency distributions were constructed.

The Item-Level Content Validity Index (I-CVI) was calculated to assess the booklet content, appearance and semantic validity. The Concordance Index (CI) value attributed to each objective of the booklet was calculated based on the level of agreement among experts, obtained by applying the formula: CI = sum of the number of agreement responses/total number of responses X 100. A minimum CI of 80% was adopted for the assessment to be considered adequate or excellent. The comparison of the CVI value with the minimum reference value was made using the binomial test. All conclusions were drawn considering the significance level of 5%.

## RESULTS

The educational booklet is composed of 52 screens, covering four thematic axes: 1) Concepts and types of biological sex, gender, gender identity, gender expression and sexual orientation; 2) LGBTphobia and the impact on LGBT+ people’s health; 3) Barriers to LGBT+ people’s health and actions to address them; 4) Policy and legislation on LGBT+ people.

The booklet was validated in terms of content and appearance by 22 judges specialized in LGBT+ health from different states (Alagoas, Bahia, Espírito Santo, Minas Gerais, Pernambuco, Piauí, Rio de Janeiro, Rio Grande do Sul, São Paulo) from different regions of Brazil. This allowed booklet assessment in different contexts, with the possibility of being applicable to other realities, based on the regional diversity of judges. Furthermore, due to the need for a multifaceted view of LGBT+ people’s health and intersectoral actions, but also in line with Fehring’s (1994) recommendation regarding the inclusion of judges with different profiles to better qualify the validity, expert judges belonged to different professional categories (11 nurses, eight psychologists, one speech therapist, one physician and one public health specialist).

The majority of judges are between 31 and 39 years old (40.9%); identify as cis men (50%); are heterosexual or homosexual (both groups with 45.5%); have graduated more than ten years ago (40.9%); hold a teaching position (45.5%); are teachers (50%); have worked as a teacher for between two and five years (54.5%); and have a master’s degree as their highest qualification (45.5%), followed by a doctoral degree (40.9%). The majority of judges participate in research groups/projects on health education, LGBT population’s health, combating LGBTphobia in healthcare services or nursing care for LGBT people (59.1%).

The booklet face validity was performed using IVATES. As indicated in Table 1, I-CVI was higher than 0.90 for all questions assessed in IVATES, indicating that all were considered adequate by judges. The binomial test was significant (p-value lower than 0.05) for all items, indicating that the I-CVI value is statistically higher than the reference value (0.80), i.e., the items assessed have validity for application.

**Table 1:** Educational booklet validity through the use of the Instrument for Validity of Appearance of Educational Technologies in Health

IVATES question	I-CVI^1^	*p* value
Q1. Illustrations are appropriate for the target audience	0.95	0.048^1^
Q2. Illustrations are clear and easy to understand	1.00	0.007^1^
Q3. Illustrations are relevant to the target audience’s understanding of the content	0.95	0.048^1^
Q4. Illustration colors are appropriate for the type of material	0.95	0.048^1^
Q5. The shapes of illustrations are appropriate for the type of material	1.00	0.007^1^
Q6. Illustrations portray the daily life of the intervention’s target audience	1.00	0.007^1^
Q7. Figure arrangement is in harmony with the text	1.00	0.007^1^
Q8. The figures used elucidate the educational material content	1.00	0.007^1^
Q9. Illustrations help to present the topic and are in a logical sequence	1.00	0.007^1^
Q10. Illustrations are in an adequate quantity in the educational material	1.00	0.007^1^
Q11. Illustrations are in an adequate size in the educational material	1.00	0.007^1^
Q12. Illustrations help to change the target audience’s behaviors and attitudes	1.00	0.007^1^
**General I-CVI**	0.98	<0.001^1^

1 *Content Validity Index*

2 Binomial test p-value (H_0_:p=0.80 x H_1_:p≠0.,80); IVATES - Instrument for Validity of Appearance of Educational Technologies in Health.

The booklet content validity was performed using IVCES. In Table 2, the I-CVI value was greater than 0.90 for all questions assessed in IVCES, indicating general adequacy of items. The binomial test was significant (p-value less than 0.05) for most items, indicating that the I-CVI value is statistically greater than the reference value (0.80). Some questions of the instrument, however, presented a non-significant p-value (p-value = 0.154), indicating that the CVI value is statistically similar to the minimum reference value (0.80).

**Table 2:** Validity of the booklet’s content using the Instrument for Validity of Educational Content in Health

Questão do IVCES	CVI	*p* value
Q1. Covers the proposed topic	1.00	0.007^1^
Q2. Suitable for the teaching-learning process	0.95	0.048
Q3. Clarifies doubts about the topic addressed	1.00	0.007^1^
Q4. Provides reflection on the topic	0.95	0.048
Q5. Encourages behavior change	0.91	0.154
Q6. Language appropriate to the target audience	0.95	0.048
Q7. Language appropriate to the educational material	0.95	0.048
Q8. Interactive language, allowing active involvement in the educational process	1.00	0.007^1^
Q9. Correct information	1.00	0.007^1^
Q10. Objective information	1.00	0.007^1^
Q11. Clarifying information	0.95	0.048
Q12. Necessary information	1.00	0.007^1^
Q13. Logical sequence of ideas	0.91	0.154
Q14. Current topic	0.95	0.048
Q15. Appropriate text size	0.91	0.154
Q16. Encourages learning	1.00	0.007^1^
Q17. Contributes to knowledge in the area	1.00	0.007^1^
Q18. Arouses interest in the topic	1.00	0.007^1^
**General I-CVI**	0.97	<0.001^1^

1 Content Validity Index

2 Binomial test p-value (H_0_:p=0,80 x H_1_:p≠0,80); IVCES - Instrument for Validity of Educational Content in Health.

The booklet items were validated with an agreement equal to or greater than 80%. The material presented an overall CVI of 0.98% for appearance and 0.97% for content, and was therefore considered valid and adequate due to its high level of agreement among experts.

The main suggestions made by experts were: rewriting the concepts of transvestism and biological sex more clearly; removing words in English for greater inclusion in the reading; correcting the information on the use of pre-exposure prophylaxis for HIV and post-exposure prophylaxis, with the inclusion of information on pre-exposure prophylaxis on demand; highlighting that trans women should not be blamed for the use of injectable silicone; and adapting some images to the information provided, such as the inclusion of the image of the combined prevention mandala on the screen that provides information on forms of sexual healthcare and prevention of sexually transmitted infections (STIs) and HIV. It should be noted that all suggestions were accepted. After the booklet was validated by experts, the material was again sent to the design professional to make the necessary adjustments to content and layout.

To characterize the personal profile, professional profile and knowledge of nurses (target audience) about LGBT+ people’s health and about the Brazilian National Policy for Comprehensive Health for Lesbian, Gay, Bisexual, Transvestite and Transgender People and qualifications for care of LGBT+ people, percentage frequencies were calculated, and frequency distributions were constructed. On average, nurses are 36.92 years old, with a standard deviation of 6.53 years. The majority identify as cis women (91.7%), heterosexual (83.3%), and graduated between 2002 and 2010 (66.7%).

Furthermore, most professionals did not receive information about LGBT people’s health during their undergraduate studies (58.3%); had a specialization/residency (66.7%); worked as a nurse and also as a nurse in primary care for between 11 and 21 years (58.3%); received information about LGBT people’s health throughout their professional career (75%); and cited “LGBT Health” in the category of main content to be addressed during an educational intervention for nurses on care for LGBT people and tackling LGBTphobia.

The booklet semantic validity was performed using SAM. Table 3 shows that the I-CVI value was equal to 1.00 for all questions, indicating that they were considered excellent/adequate by judges. The binomial test was not significant (p-value = 0.069), indicating that the CVI value is statistically similar to the minimum reference value (0.80).

**Table 3:** Booklet semantics validity using the Suitability Assessment of Materials

SAM question	I-CVI^1^	*p* value
Q1. The purpose of the illustration in relation to the text is clear	1.00	0.069^1^
Q2. Types of illustrations	1.00	0.069^1^
Q3. The figures/illustrations are relevant	1.00	0.069^1^
Q4. Illustrations have captions	1.00	0.069^1^
Q5. Layout characteristics	1.00	0.069^1^
Q6. Font size and type	1.00	0.069^1^
Q7. Subtitles are used	1.00	0.069^1^
Q8. Uses interaction	1.00	0.069^1^
Q9. The instructions are specific and give examples	1.00	0.069^1^
Q10. Motivation and self-efficacy	1.00	0.069^1^
Q11. It is similar in logic, language and experience	1.00	0.069^1^
Q12. Cultural image and examples	1.00	0.069^1^
**General I-CVI**	1.00	<0.001^1^

1 Content Validity Index

2 Binomial test p-value (H_0_:p=0,80 x H_1_:p≠0.80); SAMS - Suitability Assessment of Materials.

The booklet semantic assessment considered illustration, layout, learning stimulation/motivation and cultural suitability, obtaining a statistically significant percentage of 0.80%, therefore considered “high” in all items.

## DISCUSSION

Scientific evidence on dissemination of information related to LGBT+ health to healthcare professionals is incipient, especially in Brazil^(6,7)^. This gap motivated the creation of a digital educational booklet, which was validated by LGBT+ health experts with different academic backgrounds. The booklet configured a valuable tool to enrich the teaching-learning process by promoting debates and training educational actions aimed at planning and implementing reception and healthcare for the LGBT+ population with humanization and effectiveness.

Digital educational technologies have been increasingly used by healthcare professionals, an example of a strategy to promote the modernization and effectiveness of health practices and to contribute to improving quality of care for individuals and communities. Since the use of digital educational technologies can influence decision-making and the implementation of care actions for the population, it is imperative that the knowledge disseminated is based on evidence^(21)^.

From this perspective, the digital booklet “Knowing to care. Educational booklet on LGBT+ health for Primary Health Care professionals” was developed after a comprehensive literature review and identification of sociodemographic, educational, and cultural factors of Primary Health Care professionals and their information needs about LGBT+ people’s health. In order to ensure the attractiveness of the material and capture the reader’s attention, informative texts were produced and images and illustrations were used to reinforce the content explored.

Content includes concise information on the definitions of sexual and gender diversity, such as: biological sex, gender, gender identity, gender expression and sexual orientation, in addition to their types; the relationship between LGBTphobia and LGBT+ people’s health; barriers that are imposed on LGBT+ people in accessing health; and actions that can be taken by healthcare professionals to minimize or eliminate them. The Brazilian National Policy for Comprehensive Health for Lesbian, Gay, Bisexual, Transvestite and Transgender People and legislation on LGBT+ people provide graphic representation of the flags of lesbian, gay, bisexual and trans visibility, and regional contacts (Municipality of Recife-PE) for support and reference for LGBT+ people. All information was based on the National Policy for Comprehensive Health for Lesbian, Gay, Bisexual, Transvestite and Transgender People, and endorsed by scientific productions on the subject.

The use of different and striking colors in the booklet alludes to the rainbow, a symbol that represents, through its various colors, all forms of gender identity and sexual orientation. The adoption of the rainbow, initially by the gay community and, later, by the LGBT+ population, occurred due to three symbolic attributes: natural character (because it is a natural phenomenon and, therefore, contradicts the main pillar of homophobia, which states that relationships between people of the same sex are something that goes against human nature); universal (because the rainbow occurs all over the world as a natural phenomenon); and harmless (because it is understood as a symbol of peace, joy and tranquility). The LGBT+ rainbow flag symbolizes the movement’s gender and sexual identity politics, which are still little understood and accepted^(22)^.

Little or no knowledge about the representations that permeate the various ways of being, feeling and existing of people contributes to the promotion of various types of violence, such as verbal, emotional, psychological and physical violence. This factor denotes the importance of addressing, in the booklet, concepts related to biological sex, gender, gender identity and gender expression. Historically, especially intersex and transgender people (transvestites, transsexuals), have been stigmatized, marginalized and persecuted, due to the belief of abnormality or disorder of experiences resulting from the understanding that it is natural for the biological definition of male and female to have precise limits, such as the conception that the gender assigned at birth is the one with which the person identifies according to socially expected gender roles^(23,24)^.

Thus, transgender people experience extreme exclusion in society, where their identity is not recognized and fundamental rights, including the right to life, are not guaranteed. According to a survey by the *Grupo Gay da Bahia*, in 2023, at least 127 transgender people were murdered, acts motivated by prejudice against people who make up a socially unprotected group^(23-25)^.

Although the Health Users’ Rights Charter guarantees every Brazilian citizen universal, comprehensive and humanized access to health, LGBT+ people are vulnerable to discrimination in healthcare services, due to the heteronormative cis culture, which places transgenderism and other forms of experiencing sexuality - other than heterosexual - in the place of abnormality or deviance^(25-27)^. This fact became evident in a study^(28)^ conducted with 4,176 men who have sex with men in 12 Brazilian capitals, revealing that 9% of them suffered from LGBTphobia in healthcare services. Another study^(3)^ found that women who have sex with women, in addition to suffering discrimination related to sexual orientation, encounter greater difficulties in accessing healthcare services, such as in the case of gynecological care.

Transvestite and transsexual people face constant experiences of discrimination and communication problems due to disrespect from professionals under Decree 8,727/2016^(29)^, which guarantees the use of social name; this is considered one of the most significant complaints of poor care in healthcare services, which promotes marginalization and exclusion in health of these people^(23,27)^.

There is consistent evidence in the literature of the association of LGBTphobia with negative health outcomes in relation to cardiovascular and eating disorders, use of illicit substances, alcohol abuse, psychological stress due to discrimination (minority stress), depression, suicidal ideation, vulnerability to risky sexual behaviors for acquiring HIV, STIs, and viral hepatitis^(3,27)^.

It is extremely important for all healthcare professionals to be aware of the legal basis for the Criminalization of Homophobia, through Law 771/89, updated in 2019^(30)^, which defines any discrimination related to sexual orientation or gender identity as a crime so that they can provide clarification and support to LGBT+ people who are victims of prejudice, stigma and discrimination, in addition to disseminating information among other users of healthcare services, since, as already explained, LGBTphobia violates rights and causes social exclusion, which leads to suffering, illness and premature death for LGBT+ people.

The digital booklet developed and validated can be used as a tool for health education, either individually or collectively, as it provides adequate information to support the care of LGBT+ people in Primary Health Care. The importance of such an instrument is even greater because it aims to improve the reception and care of this population group by addressing, albeit succinctly, in a clear and simple way, the health conditions that determine them and strategies to reduce them, which are still little explored during healthcare professionals’ training and work experience.

### Study limitations

The study has a limitation in that it did not involve the participation of LGBT+ health users in the content and appearance validity and semantic assessment stages, in addition to the collaboration of professionals from other areas of training in semantic assessment, which may differ from the agreement found in other classes of healthcare professionals. This technology, however, is evidence-based and has the potential to be adapted to multidisciplinary contexts.

Although the booklet achieved an excellent content and appearance validity index among expert judges and semantic assessment by the target audience, another study limitation was that it did not assess the effects on Primary Health Care professionals’ knowledge. Further studies assessing LGBT+ health knowledge scores of primary care healthcare professionals after using the digital booklet will be necessary.

In this study, the acronym LGBT+ was used to include the group of people with a sexual orientation and/or gender identity different from what society advocates as normal, since the nomenclature used in the Brazilian National Policy for Comprehensive Health for Lesbian, Gay, Bisexual, Transvestite and Transgender People was adopted for its construction. However, although the broader acronym LGBTQIAPN+ is currently used, this study refers to the need for knowledge and discussion of the health demands of these people for inclusive health practices and care.

### Contributions to nursing, health or public policy

Addressing the most prevalent demands on LGBT+ health enables healthcare professionals to recognize, approach the topic and appropriate elements necessary for professional practice that converges with the fundamental principles of the SUS of universality, comprehensiveness and equity, through the acquisition and/or complement of knowledge that may be reflected in their various individual and team actions in healthcare services, whether preventive, curative, palliative and in health education, thus aiming to encourage research in this area.

## CONCLUSIONS

The educational booklet “Knowing to care. Educational booklet on LGBT+ health for Primary Health Care professionals”, developed according to evidence from the literature on the topic and the Brazilian National Policy for Comprehensive LGBT Health, resulted in a material validated in terms of content and appearance by evaluators with expertise in the topic. The target audience considered the educational booklet validated, through semantic assessment, which demonstrated that the developed material presents clarity through texts and illustrations, and can promote knowledge among healthcare professionals in Primary Health Care for LGBT+ people.

## Data Availability

The research data are available within the article.
