# Supplementary material for: Development and validity of an educational booklet on LGBT+ health in Primary Health Care
Source: Rev Bras Enferm. 2025 Nov 21;78(Suppl 2):e20240433. doi: 10.1590/0034-7167-2024-0433 (PMC12659914; doi:10.1590/0034-7167-2024-0433)
Supplement: Translation

# Translation: Desenvolvimento e validação de uma cartilha educacional sobre saúde LGBT+ na Atenção Primária à Saúde

**DOI:** 10.1590/0034-7167-2024-0433pt

**Authors:** Milka Gabrielle de Lira Nóbrega West, Ednaldo Cavalcante de Araújo, Cláudia Margareth de Lira Nóbrega Vilar, Maria Amanda Lima Batista, Danilo Martins Roque Pereira

**Affiliations:** I Universidade Federal de Pernambuco. Recife, Pernambuco, Brasil; II Governo do Estado de Pernambuco, Secretaria de Saúde. Recife, Pernambuco, Brasil

**Keywords:** Minorias Sexuais e de Gênero, Tecnologia Educacional, Educação Continuada, Estudo de Validação, Atenção Primária à, Saúde.

## Abstract

**Objetivos:**

desenvolver e validar uma cartilha educacional digital sobre saúde LGBT+ para profissionais na Atenção Primária à Saúde.

**Métodos:**

estudo metodológico, elaborado por meio do levantamento bibliográfico e análise do perfil sociodemográfico, educacional e cultural dos profissionais da Atenção Primária à Saúde, validação de conteúdo, aparência e semântica. Para validação, adotou-se o Índice de Validade de Conteúdo igual ou superior a 0,80, teste binomial e o *Suitability Assessment of Materials.*

**Resultados:**

a cartilha é composta por quatro eixos: conceitos sobre diversidade sexual e de gênero; LGBTfobia; barreiras à saúde de pessoas LGBT+; e políticas e legislações sobre pessoas LGBT+. Em relação à validação de conteúdo e aparência e à avaliação de semântica, a cartilha alcançou Índice de Validação de Conteúdo mínimo de 0,97.

**Conclusões:**

a cartilha foi considerada válida, podendo ser utilizada enquanto ferramenta de educação permanente de profissionais de saúde. Devem ser realizados estudos para verificar sua efetividade.

## INTRODUÇÃO

As pessoas lésbicas, gays, bissexuais, travestis, transexuais e outras formas de identidade de gênero e/ou de orientação sexual (LGBT+), por comporem um grupo que diverge das regras heteronormativas, têm sido cientificamente denominadas minorias sexuais e de gênero, e erroneamente são julgadas uniformes, ao serem desconsideradas as múltiplas e complexas experiências vivenciadas e associadas aos fatores de interseccionalidade relacionadas a elas, a exemplo de emprego, renda, escolaridade e raça/cor^(1,2)^. Embora tenham representantes em diversas esferas sociais, tais grupos enfrentam uma dinâmica coletiva de injúrias, estigmas e violências relacionadas a identidades de gênero que não correspondem às expressões, comportamentos e papéis sociais previamente designados, ou a uma orientação sexual diferente da heterossexualidade^(3)^.

Pessoas LGBT+ enfrentam diversas barreiras para acessarem cuidados em serviços de saúde, entre as quais estão as atitudes e comportamentos dos profissionais de saúde. Muitas vezes, os profissionais expressam atitudes negativas ou LGBTfóbicas. Muitos são, portanto, os obstáculos à comunicação, uma vez que não é incomum a marginalização de tal grupo populacional nos serviços de saúde devido ao uso de linguagem ofensiva, violenta por meio de palavras pejorativas, piadas, maus-tratos verbais, gestos LGBTfóbicos, utilização de pronomes e realização de perguntas que invalidam a identidade de gênero e orientação sexual, sobretudo para as pessoas travestis e transexuais que, muitas vezes, não têm sequer o direito ao nome social respeitado^(4,5)^.

Outra barreira para o acesso aos cuidados de saúde é a escassez de conhecimento e conscientização entre os profissionais de saúde sobre as necessidades específicas da população LGBT+. Tal lacuna está presente tanto nos currículos formativos quanto nos programas de educação permanente em saúde, que, frequentemente, contêm pouca ou nenhuma informação sobre orientação sexual e identidade de gênero^(6)^. Além disso, a visibilidade da temática “saúde LGBT” na literatura científica é limitada^(7)^. Consequentemente, os profissionais comumente assumem - de maneira equivocada - que as necessidades de saúde das pessoas LGBT+ são homogêneas, e, portanto, desconsideram as singularidades e nem sempre sabem rastrear adequadamente quais as vulnerabilidades impostas com mais frequência para o adoecimento e fornecer orientações adequadas sobre os cuidados de sua saúde^(2,4,5)^.

A Atenção Primária à Saúde é a porta de entrada para a assistência, e tem, enquanto uma das resoluções, a melhoria de acesso, que deve ser universal, além de prestar um cuidado integral aos usuários e às comunidades, a partir da identificação das demandas e necessidades sociais e de saúde das pessoas e grupos pertencentes à área de atuação das equipes de Saúde da Família (eSF). Tais ações e planejamentos, na maioria das vezes, no entanto, não consideram a população LGBT+ no papel de usuária dos serviços e pessoas com necessidades específicas que determinam e condicionam a situação de saúde^(8-10)^.

Os profissionais de saúde devem acolher, compreender e assistir de forma respeitosa e resolutiva as demandas específicas de saúde de pessoas LGBT+. No entanto, existe a fragilidade no cuidado a elas, ocasionada por concepções e definições históricas, e aspectos culturais, ainda impregnados de tabus e preconceitos, influenciados pela escassez de informações acerca das demandas de saúde de tais populações, que perpassam a formação acadêmica e as práticas profissionais. Devido à exposição contínua e desproporcional ao estresse relacionado ao estigma, as pessoas LGBT+ evitam buscar os serviços de saúde, ou quando procuram, não revelam a orientação sexual e/ou identidade de gênero como medida protetora para não sofrer preconceito, o que impossibilita a identificação de necessidades específicas, a realização de uma assistência resolutiva e o estabelecimento de uma linha de cuidado^(1,5,10,11)^.

Para transformar, portanto, os processos de trabalho nos serviços de saúde, nos quais o cuidado em saúde deve ser centrado nas necessidades das pessoas, é necessário que os profissionais de saúde sejam capacitados para cuidar da saúde das pessoas LGBT+, ao visar à qualidade da assistência e de vida delas. Assim, aponta-se para a necessidade de ampliar o uso de tecnologias educacionais que auxiliem os profissionais a se tornarem preparados para o acolhimento, com estabelecimento de vínculo e atenção integral no cuidado a tal população^(10,12)^.

Uma cartilha educativa voltada para o cuidado à saúde de pessoas LGBT+ pode ser utilizada como uma estratégia importante para promover o processo de aquisição, utilização e aprofundamento de conhecimentos, domínio de habilidades e desenvolvimento de atitudes e autonomia que influenciarão a tomada de decisão e a implementação de ações de promoção da saúde pelos profissionais da atenção primária, abordando, de forma sucinta, clara e simples, temas relacionados aos conceitos básicos das questões de saúde de pessoas LGBT+ e como melhor atendê-las nos cuidados primários de saúde. Assim, a cartilha pode subsidiar as práticas de cuidados de saúde na atenção primária, na perspectiva da diversidade sexual e de gênero, por buscar promover possibilidades para a construção da equidade e integralidade em saúde, princípio do Sistema Único de Saúde (SUS), e tornar as práticas em saúde baseadas na humanização.

## OBJETIVOS

Desenvolver e validar uma cartilha educacional sobre saúde LGBT+ para profissionais da Atenção Primária à Saúde.

## MÉTODOS

### Aspectos éticos

O estudo foi conduzido de acordo com as diretrizes de ética nacionais e internacionais, aprovado pelo Comitê de Ética em Pesquisa (CEP) da Universidade Federal de Pernambuco anexado à presente submissão. O Termo de Consentimento Livre e Esclarecido (TCLE) foi obtido de todos os envolvidos no estudo por meio *online*, ao utilizar a ferramenta de formulário eletrônico *Google Forms* ^®^.

### Desenho, período e etapas do estudo

Trata-se de estudo metodológico, desenvolvido no período de setembro de 2023 a maio de 2024, composto pelas seguintes etapas: 1) elaboração e submissão do projeto ao CEP; 2) busca na literatura especializada; 3) desenvolvimento do material educacional; 4) validação do material por um comitê de especialistas e avaliação pelo público-alvo. O presente estudo foi estruturado de acordo com o *Standards for QUality Improvement Reporting Excellence* 2.0^(13)^.

Na primeira etapa, foram realizadas a elaboração e submissão do projeto do estudo ao CEP. A segunda etapa envolveu a revisão de literatura baseada em artigos científicos e informações da Política Nacional de Saúde Integral às Pessoas Lésbicas, Gays, Bissexuais, Travestis e Transexuais, além de um estudo de revisão integrativa^(6)^. A terceira etapa consistiu no desenvolvimento do conteúdo e do *layout* da cartilha educacional, fundamentada nos conteúdos extraídos da literatura e dos dados sobre conteúdo e prática em saúde LGBT+ durante a formação e atividade profissional dos profissionais de saúde da atenção primária, obtidos por meio de questionário sociodemográfico semiestruturado, no qual havia a pergunta “O que você deseja aprender sobre saúde LGBT+?”. O material produzido foi encaminhado para formatação e configuração com profissional de *design* para a diagramação. Para a criação gráfica, utilizou-se o *software* Figma^®^. A quarta etapa envolveu a validação da cartilha por especialistas em saúde de pessoas LGBT+.

### Amostra; critérios de inclusão e exclusão

Foram buscados profissionais de saúde através da Plataforma *Lattes* do Conselho Nacional de Desenvolvimento Científico e Tecnológico com a utilização da palavras-chave “saúde de pessoas LGBT+” e também por meio da amostragem não probabilística tipo bola de neve, a partir da qual foram avaliados os currículos *lattes* para verificar se os profissionais atendiam aos seguintes critérios de inclusão, ao considerar os preceitos de Jasper^(14)^: possuir conhecimento/habilidade em saúde LGBT+; experiência profissional em saúde LGBT+; e *expertise* em estudos de desenvolvimento de tecnologias educacionais em saúde.

Foram convidados por e-mail 81 potenciais juízes com título de doutor, mestre e/ou especialista e com experiência profissional em saúde de pessoas LGBT+, de diferentes estados e de todas as regiões brasileiras. Desses, 58 não retornaram o contato, e um juiz não preencheu completamente os instrumentos avaliativos, dos quais resultou amostra de 22 juízes^(15)^.

Aos juízes que concordaram em participar do estudo, foram encaminhados o TCLE, a cartilha e os instrumentos para avaliação de conteúdo (Instrumento de Validação de Conteúdo Educativo em Saúde (IVCES))^(16)^ e de aparência (Instrumento de Validação de Aparência de Tecnologias Educacionais em Saúde (IVATES))^(17)^. Foi estipulado prazo de dez dias para avaliação da cartilha e preenchimento dos instrumentos. Cada tópico presente nos instrumentos continha frases afirmativas sobre os itens, e, após a análise da cartilha, os juízes poderiam avaliar o item da seguinte forma: muito adequado; adequado; parcialmente adequado; e inadequado.

Foi também incluído espaço para as sugestões a serem realizadas pelos juízes. As sugestões foram organizadas e analisadas conforme as variáveis dos instrumentos (objetivo, estrutura/apresentação e relevância da cartilha) e devidamente acatadas. Após sugestões e análise dos especialistas, realizaram-se alterações no material da cartilha educacional com o auxílio da profissional de *designer*, o que resultou na cartilha intitulada “Conhecer para cuidar. Cartilha educacional sobre saúde LGBT+ para profissionais da atenção primária à saúde”, disponível por meio do link https://www.ufpe.br/documents/39790/2852686/CARTILHA+EDUCACIONAL+CONHECER+PARA+CUIDAR.pdf/16b26753-5fe8-44e2-8911-1e141749d387

O público-alvo foi selecionado por amostragem não probabilística do tipo bola de neve, constituído^(18)^ por 12 enfermeiros que compõem as eSF. A escolha por enfermeiros no papel de público-alvo ocorreu por ser, majoritariamente, o enfermeiro o primeiro profissional de formação universitária com o qual os usuários têm contato nos serviços da atenção primária. Nos diversos campos de atuação, destaca-se a consulta de enfermagem, cuja implementação do processo de enfermagem está relacionada à coleta do histórico, realização do diagnóstico de enfermagem e do plano de cuidados adequados, a partir do entendimento do contexto social e de saúde das pessoas LGBT+. Outro campo que requer destaque é a realização, pelo enfermeiro, de práticas educativas, com a difusão de informações qualificadas, com as pessoas e comunidades nas Unidades Básicas de Saúde, escolas e em outros espaços do território adscrito das Unidades de Saúde da Família, fator contribuinte para a sensibilização e conscientização da população^(19)^.

Para a validação semântica, os enfermeiros avaliaram individualmente a cartilha digital. Foram enviados, por e-mail, um *kit* no qual continha a cartilha em formato PDF e o instrumento *Suitability Assessment of Materials* (SAM)^(20)^, para devolução em até cinco dias.

### Análise dos resultados e estatística

Para análise dos dados, foi construído um banco de dados no programa EPI INFO, versão 3.5.4, no qual foram realizadas a validação pela dupla digitação para comparação das divergências e correção dos erros dos dados obtidos pela avaliação dos juízes especialistas e do público-alvo. Após a validação, o banco foi exportado para o *software Statistical Package for the Social Sciences*, versão 18.0, onde foi realizada a análise. Para caracterizar o perfil pessoal, perfil profissional, experiência e publicação dos juízes participantes da pesquisa, e para caracterizar o perfil pessoal, perfil profissional e conhecimento dos enfermeiros acerca da saúde de pessoas LGBT+ e sobre as políticas nacionais e qualificações para atendimento de pessoas LGBT+, foram calculadas as frequências percentuais e construídas as distribuições de frequência.

O Índice de Validade de Conteúdo por Item (I-IVC) foi calculado para avaliar a validade de conteúdo, aparência e semântica da cartilha. O valor do Índice de Concordância (IC) atribuído a cada objetivo da cartilha foi calculado a partir do nível de concordância entre os especialistas, obtido por meio da aplicação da fórmula: IC = somatória do número de respostas de concordância/número total de respostas X 100. Adotou-se o IC mínimo de 80% para que a avaliação fosse considerada adequada ou excelente. A comparação do valor do IVC com o valor mínimo de referência foi feita pelo teste binomial. Todas as conclusões foram tiradas com consideração do nível de significância de 5%.

## RESULTADOS

A cartilha educacional é composta por 52 telas, abrangendo quatro eixos temáticos: 1) Conceitos e tipos de sexo biológico, gênero, identidade de gênero, expressão de gênero e orientação sexual; 2) LGBTfobia e o impacto na saúde de pessoas LGBT+; 3) Barreiras à saúde das pessoas LGBT+ e ações para seu enfrentamento; 4) Política e legislações sobre as pessoas LGBT+.

A cartilha foi validada em conteúdo e aparência por 22 juízes especialistas em saúde LGBT+ de diferentes estados (Alagoas, Bahia, Espírito Santo, Minas Gerais, Pernambuco, Piauí, Rio de Janeiro, Rio Grande do Sul, São Paulo) de diversas regiões brasileiras, o que proporcionou, a partir da diversidade regional dos juízes, a avaliação da cartilha sob diferentes contextos, com a possibilidade de ser aplicável em outras realidades. Ademais, pela necessidade do olhar multifacetado para a saúde de pessoas LGBT+ e de ações intersetoriais, mas também em alinhamento à recomendação de Fehring (1994) quanto à inclusão de juízes com perfis variados para maior qualificação da validação, os juízes especialistas pertenceram a diferentes categorias profissionais (11 enfermeiros, oito psicólogos, uma fonoaudióloga, um médico e um sanitarista).

A maioria dos juízes possui a idade de 31 a 39 anos (40,9%); identifica-se como homem cis (50%); é heterossexual ou homossexual (ambos os grupos com 45,5%); possui tempo de formação há mais de dez anos (40,9%); ocupa cargo de ensino (45,5%); é professora (50%); trabalha como docente entre dois e cinco anos (54,5%); e possui o mestrado enquanto maior título (45,5%), seguido por doutorado (40,9%). A maioria dos juízes participa de grupos/projetos de pesquisa com a temática educação em saúde, saúde da população LGBT, enfrentamento da LGBTfobia nos serviços de saúde ou cuidados de enfermagem às pessoas LGBT (59,1%).

A validação de aparência da cartilha foi realizada com IVATES. Como indicado na Tabela 1, o I-IVC foi superior a 0,90 para todas as questões avaliadas no IVATES, indicativo de que todas foram consideradas adequadas pelos juízes. O teste binomial foi significativo (valor de p menor que 0,05) para todos os itens, indicando que o valor do I-IVC é estatisticamente maior do que o valor de referência (0,80), ou seja, os itens avaliados possuem validação para aplicação.

**Tabela 1:** Validação da Cartilha Educacional por meio da utilização do Instrumento de Validação de Aparência de Tecnologias Educacionais em Saúde

Questão do IVATES	I-IVC^1^	Valor de *p*
Q1. As ilustrações estão adequadas para o público-alvo	0,95	0,048^1^
Q2. As ilustrações são claras e transmitem facilidade de compreensão	1,00	0,007^1^
Q3. As ilustrações são relevantes para compreensão do conteúdo pelo público-alvo	0,95	0,048^1^
Q4. As cores das ilustrações estão adequadas para o tipo de material	0,95	0,048^1^
Q5. As formas das ilustrações estão adequadas para o tipo de material	1,00	0,007^1^
Q6. As ilustrações retratam o cotidiano do público-alvo da intervenção	1,00	0,007^1^
Q7. A disposição das figuras está em harmonia com o texto	1,00	0,007^1^
Q8. As figuras utilizadas elucidam o conteúdo do material educativo	1,00	0,007^1^
Q9. As ilustrações ajudam na exposição da temática e estão em uma sequência lógica	1,00	0,007^1^
Q10. As ilustrações estão em quantidade adequada no material educativo	1,00	0,007^1^
Q11. As ilustrações estão em tamanhos adequados no material educativo	1,00	0,007^1^
Q12. As ilustrações ajudam na mudança de comportamentos e atitudes do público-alvo	1,00	0,007^1^
**I-IVC geral**	0,98	<0,001^1^

1 Índice de Validade de Conteúdo

2 Valor de p do teste binomial (H_0_:p=0,80 x H_1_:p≠0,80); IVATES - Instrumento de Validação de Aparência de Tecnologias Educacionais em Saúde.

A validação de conteúdo da cartilha foi realizada por meio da utilização do IVCES. Na Tabela 2, o valor do I-IVC foi maior que 0,90 para todas as questões avaliadas no IVCES, indicando adequação geral dos itens. O teste binomial foi significativo (valor de p menor que 0,05) para a maioria dos itens, indicativo de que o valor do I-IVC é estatisticamente maior do que o valor de referência (0,80). Algumas questões do instrumento, no entanto, apresentaram valor de p não significativo (valor de p = 0,154), indicando que o valor do IVC é estatisticamente semelhante ao valor mínimo de referência (0,80).

**Tabela 2:** Validação de conteúdo da cartilha por meio da utilização do Instrumento de Validação de Conteúdo Educativo em Saúde

Questão do IVCES	IVC	Valor de *p*
Q1. Contempla o tema proposto	1,00	0,007^1^
Q2. Adequado ao processo de ensino-aprendizagem	0,95	0,048
Q3. Esclarece dúvidas sobre o tema abordado	1,00	0,007^1^
Q4. Proporciona reflexão sobre o tema	0,95	0,048
Q5. Incentiva mudança de comportamento	0,91	0,154
Q6. Linguagem adequada ao público-alvo	0,95	0,048
Q7. Linguagem apropriada ao material educativo	0,95	0,048
Q8. Linguagem interativa, permitindo envolvimento ativo no processo educativo	1,00	0,007^1^
Q9. Informações corretas	1,00	0,007^1^
Q10. Informações objetivas	1,00	0,007^1^
Q11. Informações esclarecedoras	0,95	0,048
Q12. Informações necessárias	1,00	0,007^1^
Q13. Sequência lógica das ideias	0,91	0,154
Q14. Tema atual	0,95	0,048
Q15. Tamanho do texto adequado	0,91	0,154
Q16. Estimula o aprendizado	1,00	0,007^1^
Q17. Contribui para o conhecimento na área	1,00	0,007^1^
Q18. Desperta interesse pelo tema	1,00	0,007^1^
**I-IVC Geral**	0,97	<0,001^1^

1 Índice de Validade de Conteúdo

2 Valor de p do teste binomial (H_0_:p=0,80 x H_1_:p≠0,80); IVCES - Instrumento de Validação de Conteúdo Educativo em Saúde.

Os itens da cartilha foram validados em concordância igual ou superior a 80%. O material apresentou IVC global de aparência em 0,98% e, de conteúdo, de 0,97%, considerado, portanto, válido e adequado por apresentar elevado nível de concordância entre os especialistas.

As principais sugestões realizadas pelos especialistas foram: reescrever com mais clareza o conceito de travestilidade e de sexo biológico; retirar palavras em inglês para maior inclusão na leitura; corrigir as informações sobre o uso da profilaxia pré-exposição ao HIV e da profilaxia pós-exposição, com a inclusão da informação sobre a profilaxia pré-exposição sob demanda; destacar que as mulheres trans não devem ser culpabilizadas pelo uso de silicone injetável; e adequar algumas imagens às informações fornecidas a exemplo da inclusão da imagem da mandala de prevenção combinada na tela que traz sobre as formas de cuidados à saúde sexual e prevenção de infecções sexualmente transmissíveis (ISTs) e HIV. Ressalta-se que todas as sugestões foram acatadas. Após validação da cartilha pelos especialistas, o material foi novamente encaminhado à profissional de *design* para realizar os ajustes necessários quanto ao conteúdo e *layout*.

Para caracterizar o perfil pessoal, perfil profissional e o conhecimento dos enfermeiros (público-alvo) acerca da saúde de pessoas LGBT+ e sobre a Política Nacional de Saúde Integral às Pessoas Lésbicas, Gays, Bissexuais, Travestis e Transexuais e qualificações para atendimento de pessoas LGBT+, foram calculadas as frequências percentuais, sendo construídas as distribuições de frequência. Em média, os enfermeiros possuem idade de 36,92 anos, com desvio padrão de 6,53 anos. A maioria se identifica como mulher cis (91,7%), heterossexual (83,3%), e se graduou entre os anos de 2002 e 2010 (66,7%).

Ainda, a maioria dos profissionais não recebeu informações sobre saúde de pessoas LGBT durante a graduação (58,3%); possui especialização/residência (66,7%); trabalha de enfermeiro e também de enfermeiro na atenção básica entre 11 e 21 anos (58,3%); recebeu informações sobre saúde de pessoas LGBT ao longo da atuação profissional (75%); e citou a “Saúde LGBT” na categoria de principal conteúdo para abordagem durante uma intervenção educacional para enfermeiros sobre cuidados às pessoas LGBT e enfrentamento da LGBTfobia.

A validação semântica da cartilha foi realizada por meio da utilização do SAM. A Tabela 3 mostra que o valor do I-IVC foi igual a 1,00 para todas as questões, indicativos de terem sido considerados ótimos/adequados pelos juízes. O teste binomial não foi significativo (valor de p = 0,069), indicando que o valor do IVC é estatisticamente semelhante ao valor mínimo de referência (0,80).

**Tabela 3:** Validação de semântica da cartilha por meio da utilização do *Suitability Assessment of Materials*

Questão do SAM	I-IVC^1^	Valor de *p*
Q1. O propósito da ilustração referente ao texto está claro	1,00	0,069^1^
Q2. Tipos de ilustrações	1,00	0,069^1^
Q3. As figuras/ilustrações são relevantes	1,00	0,069^1^
Q4. As ilustrações têm legendas	1,00	0,069^1^
Q5. Características do *layout*	1,00	0,069^1^
Q6. Tamanho e tipo de letra	1,00	0,069^1^
Q7. São utilizados subtítulos	1,00	0,069^1^
Q8. Utiliza a interação	1,00	0,069^1^
Q9. As orientações são específicas e dão exemplos	1,00	0,069^1^
Q10. Motivação e autoeficácia	1,00	0,069^1^
Q11. É semelhante à sua lógica, linguagem e experiência	1,00	0,069^1^
Q12. Imagem cultural e exemplos	1,00	0,069^1^
**I-IVC Geral**	1,00	<0,001^1^

1 Índice de Validade de Conteúdo

2 Valor de p do teste binomial (H_0_:p=0,80 x H_1_:p≠0,80); SAMS - Suitability Assessment of Materials.

A avaliação semântica da cartilha considerou a ilustração, *layout*, estimulação/motivação do aprendizado e adequação cultural, obtendo um percentual estatisticamente significativo de 0,80%, considerado, portanto, “superior” em todos os itens.

## DISCUSSÃO

As evidências científicas sobre a disseminação de informações relacionadas à saúde LGBT+ para os profissionais de saúde são incipientes, sobretudo no Brasil^(6,7)^. Tal lacuna motivou a criação de uma cartilha educacional digital, que foi validada por especialistas em saúde LGBT+ com diferentes formações acadêmicas. A cartilha configurou uma ferramenta valiosa para enriquecer o processo de ensino-aprendizagem, ao promover debates e capacitar ações educativas voltadas para o planejamento e implementação de acolhimento e cuidados em saúde para a população LGBT+ com humanização e eficácia.

As tecnologias educacionais digitais têm sido, cada vez mais, utilizadas pelos profissionais de saúde, um exemplo de estratégia para promover a modernização e a eficácia das práticas em saúde e por contribuírem para a melhoria da qualidade do cuidado às pessoas e comunidades. Uma vez que o uso de tecnologias educacionais digitais pode influenciar a tomada de decisão e a realização de ações de cuidados à população, é imperativo que o conhecimento disseminado seja baseado em evidências^(21)^.

Sob tal perspectiva, a cartilha digital “Conhecer para cuidar. Material educativo sobre saúde LGBT+ para profissionais da Atenção Primária à Saúde” foi desenvolvida após ampla revisão de literatura e identificação de fatores sociodemográficos, educacionais e culturais de profissionais de saúde da atenção primária e de suas necessidades de informação acerca da saúde de pessoas LGBT+. A fim de garantir a atratividade do material e apreender a atenção do leitor, foram produzidos textos informativos e utilizadas imagens e ilustrações que reforçam o conteúdo explorado.

O conteúdo inclui informações concisas sobre as definições da diversidade sexual e de gênero, a exemplo de: sexo biológico, gênero, identidade de gênero, expressão de gênero e orientação sexual, além dos seus tipos; a relação da LGBTfobia e a saúde de pessoas LGBT+; as barreiras que são impostas às pessoas LGBT+ no acesso à saúde; e ações podem ser tomadas pelos profissionais de saúde para minimizá-las ou eliminá-las. A Política Nacional de Saúde Integral às Pessoas Lésbicas, Gays, Bissexuais, Travestis e Transexuais e legislações sobre as pessoas LGBT+ traz representação gráfica das bandeiras da visibilidade lésbica, gay, bissexual e trans, e contatos regionais (Município do Recife-PE) de apoio e referência às pessoas LGBT+. Todas as informações foram embasadas na Política Nacional de Saúde Integral às Pessoas Lésbicas, Gays, Bissexuais, Travestis e Transexuais, e endossadas por produções científicas a respeito da temática.

O uso de cores diversas e chamativas na cartilha faz alusão ao arco-íris, símbolo que representa, por meio das variadas cores, todas as formas de identidade de gênero e orientação sexual. A adoção do arco-íris, inicialmente por parte da comunidade gay, e, posteriormente, pela população LGBT+, ocorreu devido aos três atributos simbólicos: caráter natural (porque é um fenômeno da natureza e, assim, contraria o principal pilar da homofobia, que afirma que a relação entre pessoas do mesmo sexo é algo que vai contra a natureza humana); universal (porque o arco-íris ocorre em todo o mundo como um fenômeno natural); e inofensivo (porque é compreendido por ser símbolo de paz, alegria e tranquilidade). A bandeira do arco-íris LGBT+ simboliza a política da identidade de gênero e sexual do movimento, ainda pouco compreendida e aceita^(22)^.

O pouco ou nenhum conhecimento acerca das representações que permeiam as diversas formas de ser, sentir e existir das pessoas contribui para a promoção de variados tipos de violências, a exemplo das verbais, emocionais, psicológicas e físicas. Tal fator denota a importância da abordagem, na cartilha, de conceitos relacionados ao sexo biológico, gênero, identidade de gênero e expressão de gênero. Historicamente, sobretudo as pessoas intersexo e transgênero (travestis, transexuais), são estigmatizadas, marginalizadas e perseguidas, devido à crença de anormalidade ou transtorno de vivências decorrente da compreensão de que é natural que a definição biológica de masculino e feminino tenha limites precisos, tal qual a concepção de o gênero atribuído ao nascimento ser aquele com a que a pessoa se identifique conforme papéis de gênero esperados socialmente^(23,24)^.

Assim, as pessoas transgênero vivem a exclusão extrema na sociedade, na qual não é reconhecida sua identidade e não são garantidos direitos fundamentais, inclusive o direito à vida. Segundo levantamento do Grupo Gay da Bahia, no ano de 2023, pelo menos 127 pessoas transgênero foram assassinadas, atos motivados por preconceito contra pessoas que compõem um grupo socialmente desprotegido^(23-25)^.

Apesar de a Carta dos Direitos dos Usuários de Saúde garantir a todo cidadão brasileiro acesso universal, integral e humanizado à saúde, as pessoas LGBT+ são vulnerabilizadas em relação à discriminação nos serviços de saúde, devido à cultura cis heteronormativa, que coloca a transgeneridade e outras formas de vivenciar a sexualidade - que não a heterossexual - no lugar de anormalidade ou desviante^(25-27)^. Tal fato tornou-se evidente no estudo^(28)^ realizado com 4.176 homens que fazem sexo com homens em 12 capitais brasileiras, revelando que 9% deles sofreram LGBTfobia nos serviços de saúde. Outro estudo^(3)^ constatou que mulheres que fazem sexo com mulheres, além de sofrerem discriminação relacionada à orientação sexual, encontram maiores dificuldades de acesso aos serviços de saúde, por exemplo, no caso, de assistência ginecológica.

Pessoas travestis e transexuais enfrentam vivências constantes de discriminação e problemas de comunicação com o desrespeito por parte dos profissionais do Decreto n^o^. 8.727/2016^(29)^, que garante o uso do nome social; esta é considerada uma das queixas mais significativas de atendimento precário nos serviços de saúde, a qual promove marginalização e exclusão em saúde dessas pessoas^(23,27)^.

Há evidências consistentes na literatura da associação da LGBTfobia com desfechos negativos em saúde em relação a distúrbios cardiovasculares e alimentares, uso de substâncias ilícitas, uso abusivo de álcool, estresse psicológico por discriminação (estresse de minorias), depressão, ideação suicida, vulnerabilização a comportamentos sexuais de risco para aquisição de HIV, ISTs, e hepatites virais^(3,27)^.

Ressalta-se a extrema importância do conhecimento por parte de todos os profissionais de saúde sobre o amparo legal da Criminalização da Homofobia, por meio da Lei n^o^. 771/89, atualizada em 2019^(30)^, que define ser crime qualquer discriminação relacionada à orientação sexual ou identidade de gênero, para que forneça esclarecimentos e apoio às pessoas LGBT+ vítimas do preconceito, estigma e discriminação, além da divulgação das informações entre os demais usuários dos serviços de saúde, uma vez que, conforme já esclarecido, a LGBTfobia viola os direitos e provoca a exclusão social, o que leva às pessoas LGBT+ ao sofrimento, adoecimento e morte prematura.

A cartilha digital desenvolvida e validada pode ser utilizada no papel de ferramenta para a educação na saúde de forma individual ou coletiva, pois fornece informações adequadas para subsidiar o cuidado das pessoas LGBT+ na Atenção Primária à Saúde. A importância de tal instrumento é ainda maior, porque tem o propósito de aprimorar o acolhimento e atendimento a esse grupo populacional por meio da abordagem, ainda que sucinta, mas de forma clara e simples, de condições de saúde determinantes para elas e estratégias para reduzi-las, ainda pouco exploradas durante a formação e vivência laboral dos profissionais de saúde.

### Limitações do estudo

O estudo possui de limitação o fato de não ter contado com a participação de usuários de saúde LGBT+ nas etapas de validação de conteúdo e aparência e de avaliação de semântica, além da colaboração de profissionais de outras áreas de formação na avaliação de semântica, o que poderá divergir da concordância encontrada em outras classes de profissionais de saúde. Tal tecnologia, no entanto, é baseada em evidências, e tem o potencial para ser adaptada a contextos multiprofissionais.

Embora a cartilha tenha alcançado um excelente índice de validação de conteúdo e aparência entre os juízes especialistas e de avaliação semântica pelo público-alvo, enquanto uma outra limitação, o estudo não avaliou os efeitos no conhecimento dos profissionais de saúde da atenção primária. Estudos posteriores, que avaliem escores de conhecimento em saúde LGBT+ de profissionais de saúde na atenção primária, após a utilização da cartilha digital, serão necessários.

No presente estudo, foi utilizado o acrônimo LGBT+ com o objetivo de incluir o grupo de pessoas com orientação sexual e/ou identidade de gênero diferente do que a sociedade preconiza como normalidade, pois se adotou para a sua construção a nomenclatura utilizada de base na Política Nacional de Saúde Integral às Pessoas Lésbicas, Gays, Bissexuais, Travestis e Transexuais. No entanto, ainda que na atualidade seja utilizado o acrônimo mais amplo LGBTQIAPN+, este estudo refere-se à necessidade de conhecimento e discussão das demandas de saúde dessas pessoas para práticas e cuidados de saúde inclusivos.

### Contribuições para as áreas da enfermagem, saúde ou políticas públicas

A abordagem das demandas mais prevalentes sobre saúde LGBT+ possibilita que os profissionais de saúde reconheçam, aproximem-se da temática e se apropriem de elementos necessários à prática profissional convergente aos princípios fundamentais do SUS de universalidade, integralidade e equidade, por meio da aquisição e/ou complemento de conhecimentos que poderão refletir em suas diversas ações individuais e em equipe nos serviços de saúde, sejam elas preventivas, curativas, paliativas e em educação em saúde, visando incentivar, assim, a realização de pesquisas nessa área.

## CONCLUSÕES

A cartilha educacional “Conhecer para cuidar. Cartilha educacional sobre saúde LGBT+ para profissionais da Atenção Primária à Saúde”, desenvolvida de acordo com as evidências da literatura da temática e da Política Nacional de Saúde Integral LGBT, resultou em um material validado quanto ao conteúdo e aparência pelos juízes avaliadores com *expertise* da temática. O público-alvo considerou a cartilha educacional validada, por meio da avaliação semântica, cuja demonstração é de que o material desenvolvido apresenta clareza por meio de textos e ilustrações, podendo promover o conhecimento pelos profissionais de saúde da Atenção Primária à Saúde de pessoas LGBT+.

## Data Availability

Os dados da pesquisa estão disponíveis no corpo do artigo.
